# The Virus and the Atmosphere: Reviewing the Trajectory of Human History

**DOI:** 10.1007/s11673-023-10253-8

**Published:** 2023-08-25

**Authors:** P. Wagner

**Affiliations:** 1 Catalan Institute for Research and Advanced Studies (ICREA), SPAIN, University of Barcelona, Barcelona, Spain; 2 University of Central Asia, Bishkek, Kyrgyzstan

**Keywords:** COVID-19, Climate change, Nature, Collective capacity, Hubris

## Abstract

The article compares the COVID-19 pandemic and climate change in terms of natural characteristics of the crisis triggers as well as of socio-political responses.

A few years ago, the recently deceased Spanish novelist and essayist Javier Marías (2018) reflected in his regular column in the periodical *El País Semanal* about the ways in which the world has been transformed since the beginning of this century. He agreed with the widespread opinion that these transformations were highly worrying but then took a step back. If we move from observations about the recent past to the beginnings of the nineteenth and twentieth centuries, then our current experience pales in comparison, he said. Between 1800 and 1818, the world had gone from revolution to reaction, passing through major warfare. Between 1900 and 1918, the proud march of industrial progress, celebrated in World Fairs, had led to the mass slaughter of the First World War. Marías ominously concluded by hoping that the years 2039–2045 will not resemble the related period of the preceding century. When he wrote, neither the COVID-19 pandemic nor the Russian war on Ukraine had yet begun. Marías died on 11 September 2022 of pneumonia, according to some reports related to an earlier COVID-19 infection. His prediction, clouded in an expression of hope, has moved closer to become true.

Over the past few years, and in particular since the start of the COVID-19 pandemic, it has become common to diagnose the present time as a rapid succession of crises. The financial crisis and subsequent austerity politics; the acceleration of climate change as experienced by ever more frequent extreme weather events; the COVID-19 pandemic; and finally (until further notice) the Russian war against Ukraine. It is easy to think that humankind is on a downward trajectory on which problems accumulate and the capacity to effectively resolve them declines, or at least is not in step with problem growth.

## Disentangling Crises

But it is important to disentangle the crises, in particular in view of understanding their causes and elaborating perspectives for their resolution or further unfolding. The financial crisis and the Russian war have historical backgrounds that help understanding why they arose, but they can also be traced to specific acts and omissions that brought them about and without which they might as well not have happened. (Significantly, adding the invasion of Ukraine to the sequence of crises has made it more difficult for critical thinkers to blame capitalism for everything that goes wrong.) This is not similarly the case for climate change and the pandemic.

Even though the details of the emergence of COVID-19 remain unclear, the hypothesis that it was of conscious human making has widely been discarded. In turn, the view that its emergence is profoundly linked to our current lifestyle and global connectedness quickly gained ground in the first year of the pandemic: However, this view is not very compelling. The so-called Spanish flu of 1918–1920 (of a follow-up of which possibly Max Weber died; see Whimster 2020) and the Great Plague of the fourteenth century spread very quickly and killed large numbers of people. True, the diffusion of the former virus was sped up by war-related movements, and the latter bacterium *Yersinia pestis* travelled with the merchants along the Silk Road, but life-styles and degrees of connectedness were nevertheless very different from the present. Viruses and bacteria find their way. Viral (and bacterial) trajectories are coming and going, rising and subsiding, the latter being accelerated by appropriate countermeasures, as largely happened during the COVID-19 pandemic. Rather than having social causes, they have social impacts, at times very considerable ones. But even the social impacts cannot be derived from their viral nature; they largely depend on the context in which a virus spreads.

In contrast, humanmade climate change is a much more recent phenomenon, and it is causally related to our fossil fuel-intensive way of living. (The article draws on experiences in one world-region more than others, but its reasoning can be widened and modified to include other world-regions.) In contrast to the financial crisis and the war in Ukraine, though, it is difficult to trace climate change to specific actions and actors. In that respect, it is similar to the pandemic. However, in contrast to the pandemic, climate change is not rising and subsiding. Global warming is accelerating, and even if drastic countermeasures were taken soon, which remains unlikely, temperatures would keep rising because of the persistence of carbon dioxide (CO_2_) in the atmosphere.

After these brief disentangling reflections, we can leave the financial crisis and the war aside for the moment and focus on the crises regarding the virus and the atmosphere. The relation between the two appears ambivalent. On the one hand, the pandemic arose just after the climate crisis had begun to occupy the central place in public debate that it deserves. As a consequence, the climate crisis was moved to second rank—for understandable reasons, at least if we need to assume that public debate can always only have one central topic at a time. On the other hand, some observers pointed to the connection between the two crises—in terms of the way they impact on human beings, and in terms of the measures that can be taken to resolve them or mitigate their impact. These two issues will be pursued in what follows.

## The Very Small and the Very Big

In ecological debates, the supposed instrumental relation of human beings to nature is often considered to be the main background cause for environmental degradation and for climate change. But the precise reasoning varies and often remains quite unclear. First, there is a terminological problem: As human beings are part of nature, it would be more appropriate to speak of human beings’ relations to non-human nature. Second, and more significant for current purposes, the meaning of instrumentality would need to be clarified. Human beings depend on non-human nature for their survival, and thus they need to apply a certain degree of instrumentality, namely by making use of the “resources”—an instrumental term—that non-human nature provides. Borrowing terminology from Karl Polanyi, it is more adequate to say that human life is always—and unavoidably so—embedded in non-human nature, and then add that a conceptual disembedding has taken place in the works of certain scholars and in the practices of certain times. (One may also read the preceding phrase as recasting Bruno Latour’s [1991] reasoning about the separation of the social and the natural.) This disembedding then permitted a *more* instrumental relation to non-human nature, such as considering landscapes as objects from which resources could be extracted for human use, transgressing the “vertical frontier” of resource exploitation (Barbier 2011). But it did not alter the fundamental fact of human embeddedness in non-human nature.

Furthermore, the COVID-19 pandemic and climate change, in particular, escape any instrumental relation of human beings to non-human nature, even though the latter is often seen under this angle. This is so because neither the virus nor the atmosphere can easily be considered as an object upon which human beings can act with a purpose, for different, almost opposite reasons: The virus is extremely small and manifold and exists in and between human and other living beings and impacts on them; the atmosphere is extremely large and encompassing and is the very precondition for human existence. (Timothy Morton [2013] proposed the term “hyper-object” for phenomena such as the atmosphere, but this is an inappropriate high-jacking of the term “object” for a purpose for which it is not suited.)

Both the virus and the atmosphere have been subjected to intense human research efforts. In the latter case, the relative certainty and unusual political consensus about global warming have only been possible through decades-long investigation, data-gathering, and model-building, with new strands often initiated by outsiders (Weart 2008). In the former case, the surprising speed with which vaccines against COVID-19 were developed was due to the long tradition of virological and epidemiological research combined with parallel advances in other areas of medical science. The nevertheless existing limits of our knowledge and understanding are attributed to the complexity of the atmosphere and the variety and variability of the virus, both of which is true. Maybe more importantly, though, both the virus and the atmosphere demonstrate to us our inalienable condition of being embedded in non-human nature. The attempted disembedding of human beings from non-human nature required to think of the elements of the latter as something from which human beings can take some distance and act upon them purposefully from this distance. This is impossible for the virus and the atmosphere, even though it has not been unthinkable. They show us the limits of our knowledge and capacity for action.

## Human Action and Unintended Consequences

From their beginnings, a central concern of the social sciences has been to provide a better understanding of unintended consequences of human actions (even though the expression was coined only much later). The early social sciences were humanist throughout; they assumed that human beings have intentions and that these intentions can become causally effective on the social world through human actions. Thus, to observe a course of history that did not appear to cohere with human intentions constituted the core problem of their research agenda.

Saying this, we refer to the late eighteenth century when Enlightenment thought had boldly suggested that human beings could shape history and that this would be a history of progress. By far not everyone was convinced, though, and two alternative views arose. One of them focused on the failure to implement one’s intentions. The rhetoric of reaction, as Albert Hirschman (1991) aptly called this reasoning, maintained that attempts at improving the human condition would have no effect (“futility”), would have effects opposite to those that were intended (“perversity”), or would endanger human achievements already made (“jeopardy”). The other view also discarded grand human attempts at improving their lives but appeared to be more optimistic. Fundamentally, it held that, once certain conditions were fulfilled, one should just let go. Increase in the wealth of nations would be the unintended consequence of permitting human beings to produce and trade, of the self-regulation of markets, as it should be called later. And a reasonable public opinion would be the unintended consequence of giving free reign to the diversity of personal opinions. In this view, any collective result is not brought forth due to intentions but as the aggregate of a large number of individual human actions. Significantly, in this view of the late eighteenth century, a desirable outcome is obtained more by omission than by action, omission namely to try to intentionally steer economic or communicative processes.

Let us return from these general reflections on human history to the virus and the atmosphere. We have maintained above that a viral pandemic is not a result of human action. But it spreads more easily and with more devastating results if nothing is done to prevent that from happening. These are unintended consequences by omission, and if this omission itself is intended, then it tends to be informed by the view of the futility, perversity, or jeopardy of any human action to halt the pandemic and/or by the belief in self-regulation, called herd immunity. The examples from the COVID-19 pandemic abound.

In turn, global warming may appear to come over us as a natural disaster of giant dimensions, but it is a result of human action. By and large, though, this has not been action with that intent and purpose; hardly anyone wanted to heat up the atmosphere (for an early fictional account to the contrary, see Verne 1889). Today, therefore, anthropogenic climate change is often—and maybe increasingly—seen as the aggregate outcome of numerous uncoordinated actions. Dipesh Chakrabarty (2021, 40), for instance, maintained that humankind “stumbled” and “slid” into the Anthropocene. However, the societal trajectory of increasing fossil-fuel intensity does hardly date back longer than a century, and the key moments can be retraced in quite some detail with regard to expectations and intentions (for an attempt see Wagner 2023; forthcoming). The possibility and likelihood of dangerous global warming has also been known for a considerable—though lesser—period. But this insight was not only contested, or its significance downplayed; the guiding assumption was that there could not be any problem with “nature” that human ingenuity could not handle. Despite not having been intended, climate change is the outcome of a thinking that holds that human beings can do (almost) everything that they intend to do.

## Hubris, Self-Regulation, and Collective Intentionality

The coincidence of the COVID-19 pandemic with the rising concern about climate change has imposed new insights on humankind—or brought back old and discarded ones. Given the sociohistorical context in which it arose, it caught humankind rather unaware and unprepared. The power of the double event resides in the combination of similarities and differences between the two crises.

Even though virologists and epidemiologists knew that more pandemics would occur, many societies had left the institutions in decay that were meant to deal with them. When COVID-19 arose, it did so with such a speed and impact that (almost) the whole globe went into an emergency mode and took radical measures that hardly anyone had expected were possible. The political reaction showed that collective action and authority were necessary and had to be (re-)elaborated against the widespread but misplaced view of liberty as the permit to do whatever one pleases to do—a view that governments themselves had promoted earlier to keep the citizenry content.

In turn, climate change is a catastrophe in slow motion. It has been known for decades, and increasingly so, and it is already there. Climate change can also be experienced through rising temperatures and extreme weather events, but only if one already knows about it and can detect the signs, otherwise one can still cling to the time-honoured knowledge about the changeability and limited predictability of the weather. Serious climate action demands radical changes in social organization and life-styles in many countries, the most influential ones all among them. Until now, however, even the quickly increasing degree of certainty about a future catastrophe has not been sufficient to lead to effective action, given that the catastrophe is still in the future. Vague promises, insufficient incentives, and barely controlled restrictions remain a version of belief in self-regulation, merely a softly and unconvincingly guided one.

Given the coincidence of COVID-19 and the climate crisis over the past almost three years, it has been suggested that there is something to learn for the climate crisis from the pandemic, namely swift and radical collective action to halt a process that risks getting out of control (for my own view at the time, see Wagner 2020). This lesson would also apply to financial globalization that led to the austerity crisis; and with some qualification it also applies to letting energy and food security become dependent on the whim of aggressive tyrants.

If this lesson has not been accepted, this is at least partly due to the fact that we live in an uneasy ideological constellation, if one wants to use such a term, or we live with a radically incoherent societal self-understanding. On the one hand, there is a commitment to personal freedom that tends towards contesting collective authority, even if the latter stems from democratic deliberation. Exceptions are accepted after a dramatic event, such as in early 2020, but they need to be short-lived. On the other hand, there is the belief that human ingenuity will be able to solve all problems. The quick production of a COVID-19 vaccine may have reinforced this belief among politicians and parts of the public, but most scientists would be more prudent. Regarding the climate crisis, this belief leads to large-scale geo-engineering, one more technology that is being advertised as problem-solving even though there is no evidence of its functioning. These two attitudes or beliefs are in contradiction with each other. But they are joined in the notion that a self-regulated market-economy will generate all the “innovations” that are needed to combat emergencies and avert catastrophe. Rather, though, this attitude combines the two kinds of unintended consequences that were discussed above, namely intentions failing through being futile, perverse, or endangering achievements, and the unintended and uncontrolled aggregate outcome of numerous individual actions.

In-between, the commitment to collective authority constituted through democratic deliberation as the most appropriate means for problem-solving, especially for emergencies, is difficult to convey. Its proponents risk being accused as authoritarian, on the one side, and as wasting time in communication rather than action, on the other. However, it is the only approach that can maintain both a commitment to human agency and an understanding of the embeddedness of human beings in non-human nature. The theorem of self-regulation is not liberating, it means an abdication of human agency; and the notion of agency that disregards unintended consequences is hubris that will create its own fall.
